# Gene-corrected p.A30P *SNCA* patient-derived isogenic neurons rescue neuronal branching and function

**DOI:** 10.1038/s41598-021-01505-x

**Published:** 2021-11-09

**Authors:** Peter A. Barbuti, Jochen Ohnmacht, Bruno F. R. Santos, Paul M. Antony, François Massart, Gérald Cruciani, Claire M. Dording, Lukas Pavelka, Nicolas Casadei, Yong-Jun Kwon, Rejko Krüger

**Affiliations:** 1 Translational Neuroscience, Luxembourg Centre for Systems Biomedicine, University of Luxembourg, 4362 Esch-sur-Alzette, Luxembourg; 2 Transversal Translational Medicine, Luxembourg Institute of Health, 1445 Strassen, Luxembourg; 3 Department of Neurology, Columbia University Irving Medical Center, New York, NY 10032 USA; 4 Department of Life Science and Medicine, University of Luxembourg, 4362 Esch-sur-Alzette, Luxembourg; 5 Disease Modeling and Screening Platform (DMSP), Luxembourg Centre for Systems Biomedicine, University of Luxembourg & Luxembourg Institute of Health, 6 avenue du Swing, 4367 Belvaux, Luxembourg; 6 Parkinson Research Clinic, Centre Hospitalier de Luxembourg (CHL), 1210 Luxembourg City, Luxembourg; 7 Institute of Medical Genetics and Applied Genomics, University of Tübingen, Tübingen, Germany; 8 NGS Competence Center Tübingen, Tübingen, Germany; 9 Department of Oncology, Luxembourg Institute of Health, 3555 Dudelange, Luxembourg

**Keywords:** Neuroscience, Molecular neuroscience, Neurological disorders, Stem-cell research, Ageing, Stem-cell differentiation

## Abstract

Parkinson’s disease (PD) is characterised by the degeneration of A9 dopaminergic neurons and the pathological accumulation of alpha-synuclein. The p.A30P *SNCA* mutation generates the pathogenic form of the alpha-synuclein protein causing an autosomal-dominant form of PD. There are limited studies assessing pathogenic *SNCA* mutations in patient-derived isogenic cell models. Here we provide a functional assessment of dopaminergic neurons derived from a patient harbouring the p.A30P *SNCA* mutation. Using two clonal gene-corrected isogenic cell lines we identified image-based phenotypes showing impaired neuritic processes. The pathological neurons displayed impaired neuronal activity, reduced mitochondrial respiration, an energy deficit, vulnerability to rotenone, and transcriptional alterations in lipid metabolism. Our data describes for the first time the mutation-only effect of the p.A30P *SNCA* mutation on neuronal function, supporting the use of isogenic cell lines in identifying image-based pathological phenotypes that can serve as an entry point for future disease-modifying compound screenings and drug discovery strategies.

## Introduction

Parkinson’s disease (PD) is a neurodegenerative disease with no current causative treatment. It is one of the world’s fastest growing neurological disorders^1^, with a global burden expected to reach 17 million by 2040^1,2^. PD has two defining neuropathological features; the first is the degeneration of the A9 dopaminergic neurons of the substantia nigra pars compacta (SNc). It is still unclear why specifically these neurons degenerate, although the unique cellular architecture of these dopaminergic nigral neurons including their large complex arborisation, high bioenergetic demands and resultant oxidative stress, combined with neuroanatomical telencephalization have been hypothesised among the reasons for this specific vulnerability^3–5^. Yet to provide balance, an alternative and widely-held viewpoint is that PD is a prion-like disorder^6,7^. Simply, the origin of PD pathogenesis remains unclear. The formation of intra-cytoplasmic inclusion bodies (Lewy Bodies, LBs) in the dopaminergic neurons that remain is the second defining neuropathological feature of PD. These LBs contain a high concentration of lipids, crowded organelles and are immunopositive for alpha-synuclein^8,9^, a protein found ubiquitously in neurons that is localised to the pre-synaptic terminal^10^.

Rare and highly penetrant point mutations in the *SNCA* gene, which encodes the alpha-synuclein protein leads to the autosomal dominant form of PD at: p.A53T^11^, p.A30P^12^, p.E46K^13^, p.G51D^14^, and p.A53E^15^. Increased levels of physiological alpha-synuclein caused by multiplications of the *SNCA* locus by duplication^16,17^ or triplication^18^ also lead to familial PD in a dose-dependent way, with the triplication having more severe clinical symptoms and faster disease progression than the duplication^19^. Moreover, genome wide association studies (GWAS) have identified single nucleotide polymorphism (SNP) genetic variants in *SNCA* as risk factors in sporadic PD due to modulation of alpha-synuclein expression^20–22^.

Patient-derived iPS cells, first described by Yamanaka^23^, together with targeted genome editing tools, such as clustered regularly interspaced short palindromic repeats (CRISPR)-Cas-associated nucleases (CRISPR-Cas9)^24–26^, have enabled patient-derived cells carrying pathogenic mutations to be corrected which allows the specific effect of the pathogenic mutation to be assessed against its isogenic control. Genome editing is a technically challenging procedure and refers to editing one or more cells in a colony composed of multiple cells. Not all cells will be edited, and fluorescent reporters, antibiotic resistance or a combination of both, are used to enrich and/or sort out the edited cells to generate the isogenic cell line^27–29^. Previously, we showed that with the aid of high-content screening (HCS) technology we were able to generate multiple single-cell gene-corrected patient-derived iPS cell clones from a PD patient harbouring the pathogenic p.A30P mutation in *SNCA*^30^. The advantage of having single-cell clones is that it provides the guarantee that every cell in that colony and expanded cell line is edited and gene-corrected. Without this approach, it remains quite possible that the cellular composition of an iPS colony will contain a mixture of edited and unedited cells.

In our prior study, we generated and characterised multiple gene-corrected single-cell iPSC clonal lines^30^. In this follow-up study, we have performed functional characterisation of two gene-corrected clones and the founder cell line, where only significant differences of both gene-corrected clones against the patient cell line will be interpreted as the pathological effect of the p.A30P *SNCA* mutation. We have assessed complex 3D neuronal networks of ventral midbrain dopaminergic (vmDA) neurons by segmenting the neurons immunopositive for tyrosine hydroxylase (TH), the rate limiting enzyme in the biosynthesis of dopamine. We have identified that irrespective of neuronal abundance, the patient-derived neurons carrying the A30P mutation show decreased neurite complexity with reduced neurite branching in the vmDA neurons. We assessed the neuronal connectivity using multi-electrode array (MEA) technology and find reduced neuronal function in the patient neurons carrying the p.A30P mutation in *SNCA*. Furthermore, we also find that these vmDA neurons display a reduced mitochondrial respiration, a clear energy deficit shown by loss of ATP, reduced mitochondrial membrane potential (MMP) and a high level of mitochondrial superoxide. In addition, we treated these neurons using rotenone as an environmental toxin model for PD and found that the neurons carrying the A30P mutation in alpha-synuclein have a specific reduction in neuronal viability compared to both gene-corrected iPS-derived controls. Furthermore, changes in gene expression support an impairment of lipid metabolism in the neurons carrying the pathogenic alpha-synuclein. Together, our results support the use of isogenic cell lines to model PD pathophysiology, identifying an image-based neuronal phenotype and associated functional phenotypes from the endogenous expression of the pathogenic p.A30P *SNCA* mutation, having implications for future disease modifying screening strategies.

## Results

### Clinical phenotype of p.A30P *SNCA* patient

The pedigree of this family, previously published shows the affected patient (IV, 5—black arrow)^12,31^. These affected familial individuals carry an autosomal dominant heterozygous mutation in c.88G>C *SNCA* that translates the pathogenic A30P form of the alpha-synuclein protein. The patient concerned in this study is a right-hand dominant individual with an age of disease onset at 55 years with initial symptoms of extrapyramidal rigidity and bradykinesia dominant on the right side. The time between first symptoms to diagnosis was 3 months. The patient showed/presented with a good response to l-Dopa and was treated with Deep Brain Stimulation (DBS) in 2005 due to emerging motor fluctuations. The disease duration from diagnosis to clinical examination detailed in Supplementary Table 1 was 13 years with the age of assessment at 68 years. The patient had 8 years of formal education up to secondary school. The dopaminergic medication at the time of the examination in ON state: Madopar p.o. 125 mg ½-1/4-½-½, Madopar p.o. Depot 125 mg 0-0-0-1, calculated LEDD (L-DOPA equivalent daily dose) 250 mg/day. The medication with central effect at the time of examination: Venlafaxin p.o. 150 mg 0-0-0-1. The patient was examined by a specialized neurologist using UPDRS score that is shown in the supplementary material (Supp. Table 1) (N.B. This is the older version of the UPDRS not the MDS-UPDRS). The clinical phenotype of the motor and non-motor symptoms is found in the supplementary material (Supp. Table 2).

### Cellular characterisation including morphometric analysis of neuronal network reveals neuritic pathology with specific reduction of branchpoints in dopaminergic neurons in the p.A30P *SNCA* patient

**Figure 1:**
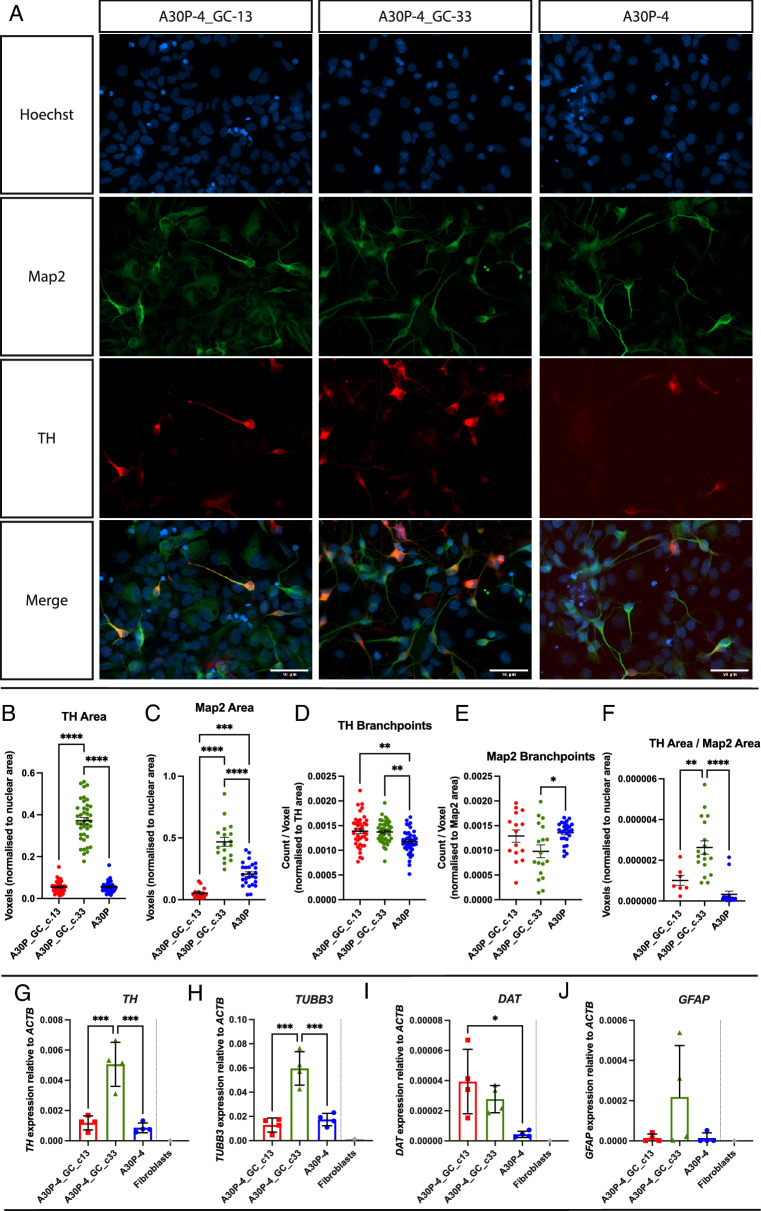
Morphometric analysis of TH network in d30 neurons. (**A**) Representative image of a maximum intensity projection of a Z-stack fluorescently labelled with Map2 and TH antibodies. Images taken using a ×25 objective, scale bar is 50 μM. Identification and quantification of parameters: (**B**) TH Area, (**C**) Map2 Area, (**D**) TH Branchpoints, (**E**) Map2 Branchpoints and (**F**) TH Area/Map2 Area were determined. Four biological replicates were used for the analysis with a minimum of six Z-stacks analysed by replicate. The graphs displayed in columns show data points that refer to the average data per Z-stack. Relative quantification in the patient-derived neurons of: (**G**) *TH*, (**H**) *TUBB3*, (**I**) *GFAP* and (**J**) *DAT*. Each data point refers to 4 independent biological replicates. For all statistical analyses, an ordinary one-way ANOVA was performed with Tukey’s post-hoc multiple comparison test. Graphs (**B**–**F**) were plotted as mean ± SEM; graphs (**G**–**J**) were plotted as mean ± SD. *p < 0.05, ** p < 0.01, ***p < 0.001 ****p < 0.0001.

In prior work, we had used CRISPR-Cas9 technology to gene-correct the c.88G>C, p.A30P *SNCA* mutation in the patient iPSCs^30^. Two single-cell patient-derived gene-corrected isogenic iPSC clones were fully characterised with vmDA neurons generated, characterised, and quantified^30^. To perform detailed neuronal network analysis, Z-stacks were acquired using confocal microscopy, and neuronal segmentation, skeletonisation and identification of morphological parameters were performed (Supp. Fig. 1) as we have previously described^32,33^. Fluorescently labelled antibodies specific for the mature neuronal marker, Microtubule Associated Protein 2 (MAP2) and the dopaminergic marker, tyrosine hydroxylase (TH) were used with a representative image of each cell line shown (Fig. 1A).

Previously we found there was no significant difference in the amount of neurons-labelled with the pan-neuronal marker TUJ1, generated between the cell lines carrying the p.A30P alpha-synuclein mutation and two gene-corrected control lines, yet the gene-corrected clone 33 cell line generated a higher number of TH positive neurons^30^. We again find significant inter-clonal variation between the two gene-corrected clones in terms of TH area (Fig. 1B) and MAP2 area (Fig. 1C) with gene-corrected clone 33 having a significantly greater proportion of TH and MAP2 neurons. Normalising to consider cellular density and differentiation propensity we identified a specific reduction in TH branchpoints (Fig. 1D), a newly identified phenotype specific to vmDA neurons carrying the p.A30P alpha synuclein protein compared to neurons generated from both isogenic gene corrected clones. Interestingly, neurons carrying the A30P mutation were able to generate an equal or greater number of MAP2 positive branchpoints (Fig. 1E), although had a reduced proportion of dopaminergic neurons to mature neurons (Fig. 1F).

In terms of differentiation characterisation, we observed differences in gene expression for *TH* (Fig. 1G), *TUBB3* (Fig. 1H) and *GFAP* (Fig. 1I) highlighting the variability within the cellular patient-derived cultures which are not specific to the p.A30P *SNCA* mutation. However, against both gene-corrected clones we found reduced expression of the *DAT* (*SLC6A3*) the gene encoding Dopamine Active Transporter (Fig. 1J).

To perform a comprehensive investigation of the composition of the cellular population we performed morphometric network analysis using fluorescently labelled antibodies against Tuj1 (Supp. Fig. 1), a neural stem cell marker Nestin (Supp. Fig. 3) and two markers of astrocytes—Glial Fibrillary Acidic Protein (GFAP) and Vimentin (Supp. Fig. 4). Additionally, we compared in RNA-seq data the gene expression levels of specific glial lineage markers, *GFAP*, *VIM*, *AQP4*, *S100B* and *SLC1A3* (*EAAT1*) (Supp. Fig. 4). Taken together, we find no presence of reactive astrocytes in our culture, a smaller but sporadic presence of astrocytes, and an elevated expression of radial glia progenitor cells.

### Multi-electrode arrays (MEAs) show reduced neuronal function in p.A30P *SNCA* patient

**Figure 2:**
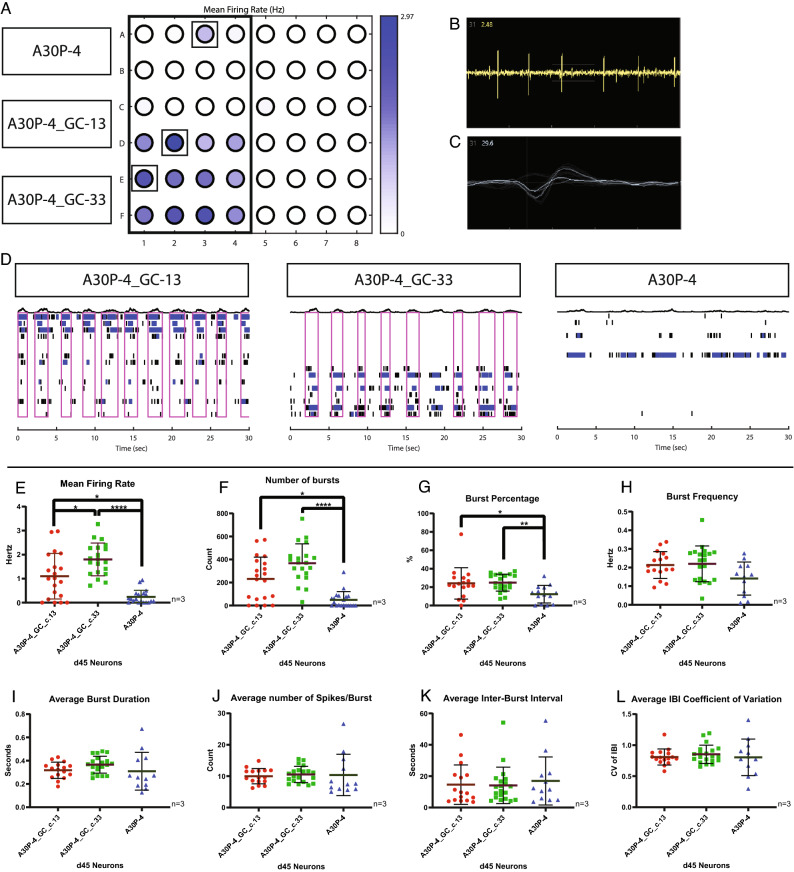
Multi-electrode array neuronal recording of d55 neurons. (**A**) Representative image showing the plate layout of recorded neurons with a heat map displaying the mean firing rate. (**B**) Representative example of a Waveform profile, (**C**) Representative example of a Spike plot, (**D**) Representative image of a raster plot profile, taken from the well highlighted in (**A**). Spike and burst metric parameters identified from the MEA recording: (**E**) Mean Firing Rate, (**F**) Number of Bursts, (**G**) Burst Percentage, (**H**) Burst Frequency, (**I**) Average Burst Duration, (**J**) Number of Spikes/Burst, (**K**) Average Inter-Burst Interval, and (**L**) Average Inter-Burst Interval (IBI) Co-efficient of Variation. Three biological replicates were used in the analysis with a minimum of six technical replicates per recording. The graphs displayed in columns show individual values that refer to the average data of each well, with each well composed of 16 electrodes. For all statistical analyses, a non-parametric Kruskal–Wallis test was performed using the Dunn’s post hoc multiple comparison test. All graphs were plotted as mean ± SD. *p < 0.05, **p < 0.01, ****p < 0.0001.

After we had established that the neurons carrying the p.A30P alpha-synuclein mutation had a reduction in branchpoints, a measurement of synaptic connections, we hypothesised that this would influence neuronal function. We used extracellular multi-electrode arrays (MEAs) to measure neuronal activity in-vitro. A representative example of the seeded neurons on the MEA device is shown (Fig. 2A), where a heat-map detecting mean firing rate is the measurable parameter. An example of a raw voltage recording of a single electrode capturing a neuronal spike is shown (Fig. 2B) with a representative image of a digitally generated waveform profile displaying a neuronal action potential shown (Fig. 2C). A representative raster plot profile from a neuronal recording is displayed showing that each cell line exhibits functional active neurons (Fig. 2D). The neurons carrying the A30P mutation have reduced neuronal function compared to both gene-corrected clones with decreased mean firing rate (Fig. 2E), decreased number of synchronised spiking neurons “bursts” (Fig. 2F), and a decreased percentage of bursts (Fig. 2G). Additional parameters of neuronal function generated by the analysed neuronal recording of burst frequency, average burst duration, average number of spikes/burst, average inter-burst interval (IBI), and average IBI coefficient of variation (Fig. 2H–L) show no difference between the neurons carrying the A30P mutation and the gene-corrected neurons without the A30P mutation.

### Neurons carrying the p.A30P *SNCA* mutation have reduced mitochondrial function

**Figure 3:**
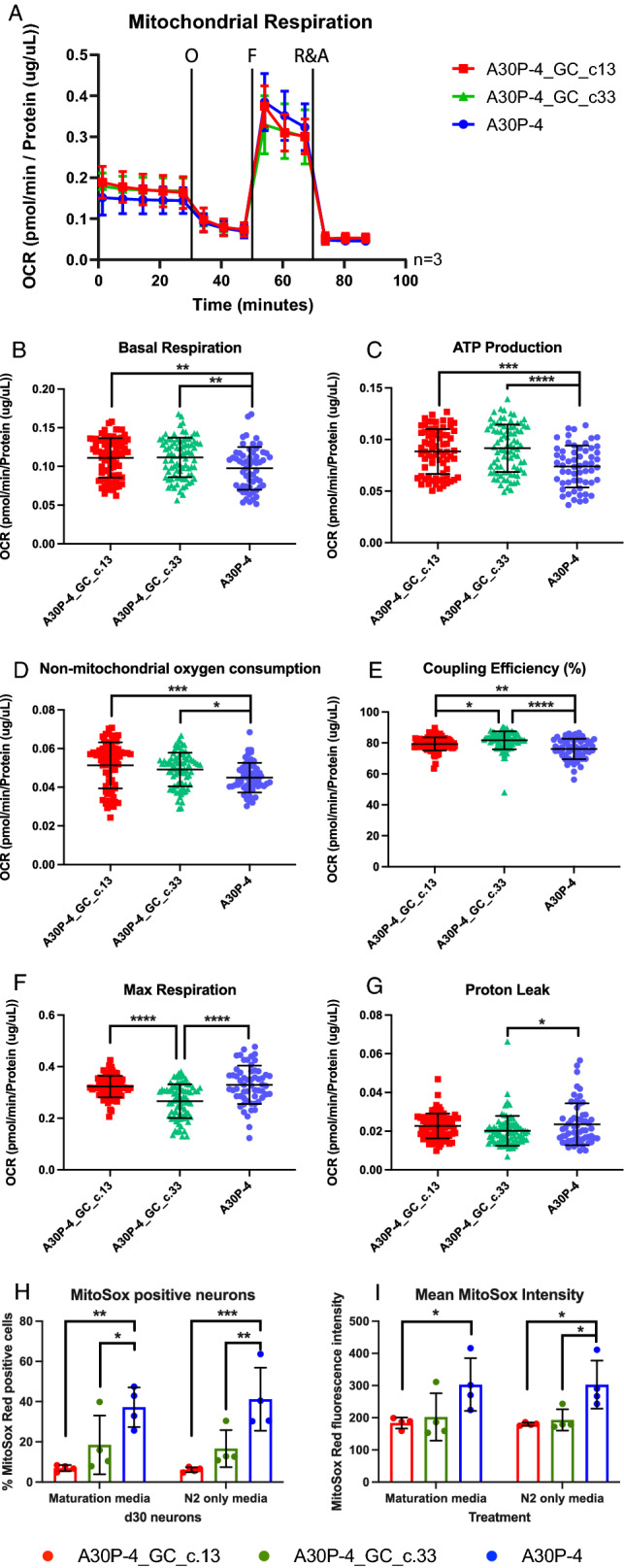
Mitochondrial dysfunction in neurons carrying the p.A30P SNCA mutation. (**A**) Bioenergetic profile showing oxygen consumption rate (OCR) of patient-derived neurons following a mitochondrial stress test under basal conditions and following the treatments of the ATP synthase inhibitor oligomycin (O, 1 µM), the oxidative phosphorylation uncoupler FCCP (F, 500 nM), and the electron transport chain inhibitors rotenone (Complex I) and antimycin A (Complex III) (R&A, 10 µM). The cumulative OCR profile is shown in ventral midbrain neurons differentiated for 30 days (n = 3). The rates of (**B**) Basal Respiration, (**C**) ATP production, (**D**) Non-mitochondrial respiration, (**E**) Coupling efficiency, (**F**) Maximum Respiration and (**G**) Proton Leak are shown. Each data point refers to individual values taken from a minimum of 12 replicates from 3 independent biological replicates. Mitochondrial reactive oxygen species measured by flow cytometry of (**H**) MitoSOX Red positive cells and (**I**) MitoSOX Red mean fluorescence intensity in typical “maturation” culture medium or with N2 media (without B27, trophic factors and antioxidants) for 4 h (n = 4). Each data point refers to 4 independent biological replicates. For all statistical analyses, an ordinary one-way ANOVA was performed using the Tukey post-hoc multiple comparison test. All graphs were plotted as mean ± SD. *p < 0.05, ** p < 0.01, ***p < 0.001 ****p < 0.0001.

The results obtained from the MEA recording show reduced neuronal function in the A30P patient neurons. Neurons are an energy intensive cell type requiring vast amounts of ATP for synaptic function that is primarily met via oxidative phosphorylation by the mitochondria. In order the determine the effect of the A30P alpha-synuclein mutation on mitochondrial respiration we used the Seahorse XFe96 extracellular flux assay to assess the cellular bioenergetics of these mutant neurons compared to the gene-corrected controls. A mitochondrial stress test was performed with direct measurement of the oxygen consumption rate (OCR). Mitochondrial stressors: oligomycin, FCCP, antimycin A and rotenone were added to the neurons to assess different respiratory parameters with the merged profile plot of three biological replicates is shown (Fig. 3A). The neurons carrying the p.A30P mutation, when assessed against both gene-corrected controls had a significantly reduced mitochondrial respiration at basal levels (Fig. 3B), reduced capability to generate ATP (Fig. 3C), a significant reduction in non-mitochondrial respiration (Fig. 3D) and coupling efficiency (Fig. 3E). Compared to both gene-corrected isogenic controls there was no significant difference in maximum respiration (Fig. 3F) and proton leak (Fig. 3G).

To control for the influence of the genetic background we assessed the bioenergetic profile of neurons directly differentiated from two different iPS clones carrying the A30P mutation, which we have previously characterised^34^. Comparing these pathological neurons against neurons from an age- and gender-matched non-PD control^35^, we find that the bioenergetic profile of both clonal cell lines carrying the A30P alpha-synuclein mutation align, with mitochondrial respiration and ATP production significantly reduced compared to the control. To further investigate the loss of ATP, we assessed the mitochondrial membrane potential (MMP) of the neurons containing the A30P mutation against gene-corrected neurons, and to control for the genetic background, a gender-matched non-PD control that we have also previously characterised^32^, finding that in the pathological neurons MMP is reduced (Supp. Fig. 5).

As another measure of mitochondrial function, we measured the generation of mitochondrial superoxide under basal and starvation conditions. In both conditions there was a significant increase in the relative number and intensity of mitochondrial superoxide in the neurons carrying the p.A30P alpha-synuclein mutation compared to both gene-corrected controls (Fig. 3H–I).

### Neurons carrying the p.A30P *SNCA* mutation have reduced energy with decreased neuronal viability following complex I inhibition

**Figure 4:**
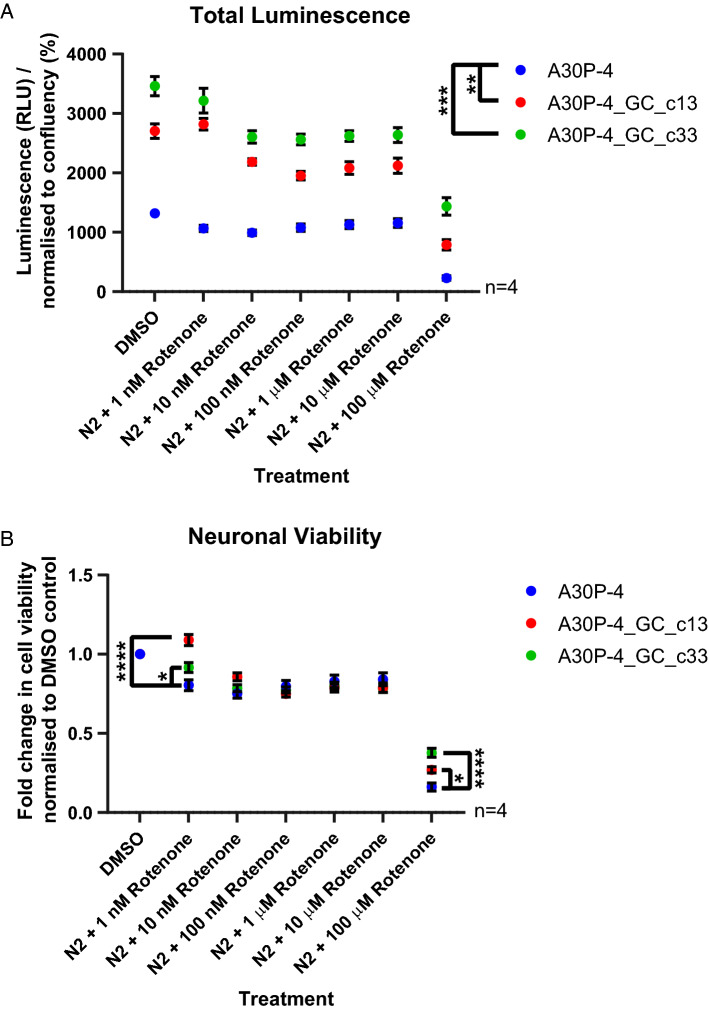
Neuronal viability after rotenone treatment. (**A**) Total luminescence profile of the patient-derived neurons using the CellTiter-Glo Luminescent Cell Viability Assay with increasing concentrations of rotenone treatment performed over 16 h. The neurons were differentiated for 40 days, a minimum of eight technical replicates were used per analysis with four biological replicates performed. For statistical analysis, an ordinary one-way ANOVA was performed using the Tukey post-hoc multiple comparison test. (**B**) Fold change in cell viability of patient-derived neurons normalised to DMSO control. A two-way ANOVA was used with a Turkey’s post-hoc multiple-comparison test. All graphs were plotted as mean ± SEM. *p < 0.05, **p < 0.01, ***p < 0.001 ****p < 0.0001.

Having identified specific functional and mitochondrial impairments in the neurons carrying the p.A30P SNCA mutation, we hypothesised that these neurons would be specifically vulnerable to toxic insult. To assess this we used rotenone, a complex I inhibitor that leads to the selective degeneration of TH neurons^36,37^. It is noticeable that compared to both gene-corrected clones the total luminescence, a measurement of cellular ATP in the viable neurons, although variable is significantly reduced in the patient neurons carrying the A30P mutation (Fig. 4A). Treatment with increasing concentrations of rotenone lead to a reduction in ATP and thereby a reduction in cellular viability (Fig. 4B). The neurons expressing the p.A30P SNCA mutation show a specific vulnerability to treatment with 1 nM of rotenone leading to a significant fold decrease in cellular viability compared to both gene-corrected controls. There is no fold change in neuronal viability between patient and gene corrected controls at concentrations between 10 nM and 10 μM. At the concentration of 100 μM of rotenone, there is a significant fold decrease in cellular viability in the neurons expressing the p.A30P SNCA mutation compared to both gene-corrected controls. Our results indicate that 100 μM of rotenone is the concentration where the majority of neurons are no longer viable, whereas the concentration of 1 nM of rotenone is sufficient to identify a selective fold change in cellular viability in differentially vulnerable neurons carrying a pathogenic A30P mutation in alpha-synuclein.

### Gene expression analysis identifies alterations in lipid metabolism

**Figure 5:**
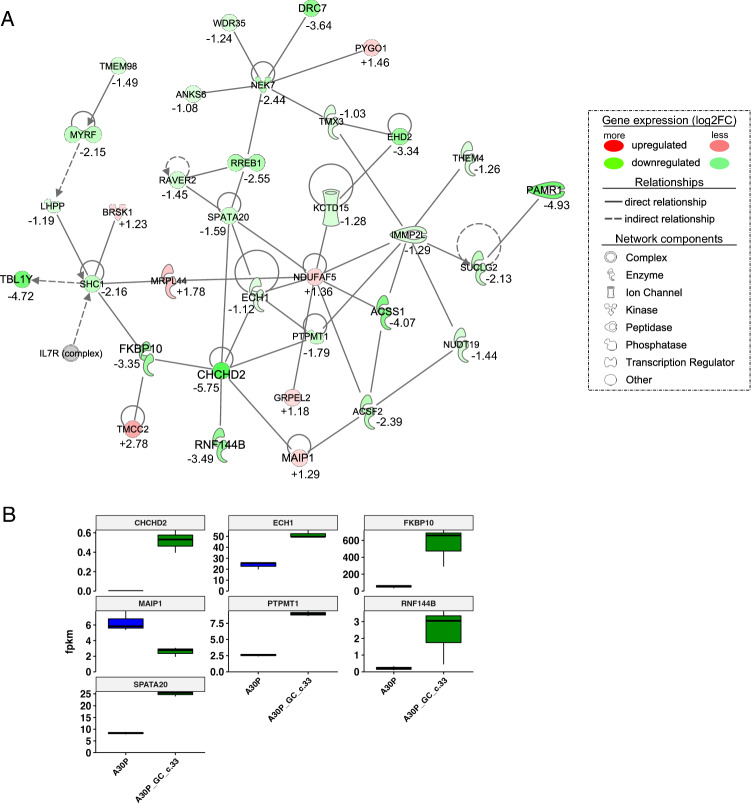
Functional analysis of gene expression networks performed by Ingenuity Pathway Analysis (IPA). (**A**) Changes in genes associated with lipid metabolism. Genes in green are upregulated, and in red downregulated in respect to the patient neurons compared to the gene-corrected isogenic control (**B**) Gene expression levels based on RNA-seq of the genes associated with lipid metabolism.

For a deeper understanding of gene expression in neurons expressing the p.A30P *SNCA* mutation against the gene-corrected neurons expressing wild-type *SNCA,* we performed RNA sequencing on RNA isolated from 45-day old neurons. We find a total of 3713 genes differentially expressed between mutant and gene corrected cultures (with a FDR cut-off of < 0.05 and a log2fold change of ± 2). Using the pathway analysis tool Ingenuity Pathway Analysis (IPA) we found that the top-ranked network with the greatest differences between patient and isogenic control to be associated with lipid metabolism (Fig. 5A). It is notable that several of the proteins coded by genes within this network: *MRPL44*, *TMX3*, *ECH1*, *PTPMT1* are located at or associate with the mitochondrial associated ER membranes (MAM), functional domains where wild-type alpha-synuclein is localised^38^.

Changes in differential gene expression of the lipid metabolism network identified the Parkinson’s risk gene *CHCHD2* (PARK 22) as significantly downregulated in the neurons carrying the p.A30P *SNCA* mutation compared to the gene-corrected isogenic control. Mutations in *CHCHD2* are associated with mitochondrial dysfunction by reduced mitochondrial respiration^39,40^. Although we find the relative gene expression of *CHCHD2* to be low (fpkm < 1) we find considerable gene expression differences in the intermediate predicted interaction partners *ECH1*, *PTPMT1* and *FKBP10* (Fig. 5B).

It is of note that both transcriptional regulators identified by this lipid metabolism network (*MYRF* and *RREB1*) are reduced in the patient neurons (Supp. Fig. 7). Furthermore, we find the Brain Specific Kinase 1 (*BRSK1)*, also known as SAD-B kinase significantly elevated in the pathological neurons carrying the p.A30P *SNCA* mutation. BRSK1 associates with lipid rafts, is an interaction partner of alpha-synuclein and phosphorylates tau^41,42^, BRSK1 has been previously identified as a blood-based biomarker for the early detection and diagnosis of PD^43^.

## Discussion

Parkinson’s disease affects many neuronal and non-neuronal cellular populations other than the innervating striatal A9 dopaminergic neurons^44^. Why this specific subtype of dopaminergic neurons in the midbrain is more affected than others and why they degenerate first is a question occupying various hypotheses elsewhere reviewed^3–6,45^. Although, the physiological role(s) of alpha-synuclein is unclear and is a much debated topic^46^, one of the generally accepted physiological functions of alpha-synuclein is the regulation of synaptic vesicle endocytosis^47,48^, with synaptic dysfunction long-being associated with PD^49,50^.

Our imaged-based morphometric analysis shows that dopaminergic neurons specifically have reduced neurite branching and connectivity. Gene expression analysis also identified that the pathogenic neurons have a specific reduction in *DAT*, which aligns our study with previous pathological studies that identify DAT depletion in the striatum of PD patients^51,52^. Taken together, these findings fit with the specific vulnerability of the DA neuron. A general assessment of neuronal function using MEAs has also found that these pathological neurons are functionally impaired in terms of neuronal spike and burst release kinetics. Our results fit with a recent study of patient-derived isogenic dopaminergic neurons carrying the p.A53T *SNCA* mutation that found neuritic pathology when compared against its isogenic control^53^. Taken together, we can consider that pathogenic alpha-synuclein facilitates neuritic impairment via synaptic dysfunction.

Underpinning all of this is energy. Dopaminergic neurons exhibit a characteristic autonomous pacemaking activity, continuous cycles of synaptic vesicle recycling and the packaging of dopamine within those synaptic vesicles. All of which, require copious amount of energy and make the dopaminergic neurons specifically vulnerable when in an energy debt. Improperly packaged cytosolic dopamine is subject to oxidation and can lead to increased levels of oxidant stress with pathogenic downstream affects^54^. We find that compared to both gene-corrected isogenic control cell lines, the neurons that express p.A30P alpha-synuclein have a severe energy deficit with reduced basal respiration, reduced non-mitochondrial oxygen consumption, lower levels of ATP production per cell, reduction in MMP and increased levels mitochondrial reactive oxygen species (ROS) detected by increased mitochondrial superoxide. Our findings align with another study that gene-corrected the patient-derived p.A53T *SNCA* mutation assessing iPS-derived dopaminergic neurons, in which deficits in mitochondrial respiration were identified as a PD phenotype^55^. Moreover, we find cellular viability reduced in the neurons carrying the pathogenic p.A30P alpha-synuclein variant compared to the gene-corrected isogenic controls. We have furthermore, defined and established that a concentration of 1 nM rotenone, an environmental pesticide and well-established toxin specific to mitochondrial electron transport chain complex I in dopaminergic neurons^36,37^, is sufficient to significantly reduce neuronal viability in our cellular model. The identification of these neurotoxic parameters has potential implications for high-throughput disease modelling and drug discovery research by identifying screening parameters to identify vulnerable neurons, before the implementation of subsequent rescue strategies.

A recent transcriptomic study on two independent case–control cohorts has identified lipid oxidation, in addition to pathways related to the ER and unfolded protein response as the top differential gene expression signature of the PD prefrontal cortex, once RNA post-mortem degradation and cell heterogeneity is taken into account^56^. To support this putative mechanism, pathway analysis on our own RNA-seq data found lipid metabolism function to be the network with highest differential expression between neurons carrying the p.A30P *SNCA* mutation compared to the isogenic control. Furthermore, we find reduced expression of ACSS1 and ACSF2, two mitochondrial acetyl-CoA synthetase enzymes implicate dysregulation of pyruvate metabolism, which has already been implicated in PD^57^, where the metabolic flexibility to provide ATP is maintaining the interplay between mitochondrial oxidative phosphorylation, glycolysis, the uptake of lactate and pyruvate entry into the mitochondria^58^. Disruption to any of these pathways can alter glucose metabolism and reduce oxidative phosphorylation, which we find under basal conditions in our cellular model of PD. Furthermore, previous studies have found under glucose deprivation, dopaminergic neurons have shown reduced spontaneous firing and membrane depolarisation^59^, cytosolic aggregation of alpha-synuclein and reduced dopaminergic uptake leading to dopaminergic neuronal death^60^. Additionally, we find reduced expression of the mitochondrial phosphatase *PTPMT1*, which is essential for the biosynthesis of cardiolipin, a phospholipid contributing up to 20% of the inner mitochondrial membrane that is crucial for proper function of the electron transport chain and other mitochondrial signalling pathways^61^. Loss of PTPMT1 was found to dramatically reduce the biosynthesis of cardiolipin, reducing mitochondria respiration and generating abnormal mitochondrial morphology^62^. Consequently, dysregulation of energy metabolism can be considered a mechanism underpinning PD.

Our study is the first functional neuronal analysis in gene-corrected isogenic cell lines from a PD patient harbouring the pathogenic p.A30P mutation in the *SNCA* gene. Only a small number of studies have generated patient-derived gene corrected isogenic cell lines harbouring pathogenic mutations in the *SNCA* gene^30,53,55,63,64^. Assessment of the endogenous p.A53T SNCA expression in dopaminergic neurons against their isogenic gene corrected control have identified mitochondrial dysfunction and apoptosis from increased basal levels of oxidative and nitrosative stress^55^, reductions in neurite length and complexity^53^, and impaired mitochondria dynamics^64^. Upregulation of ER stress and activation of the unfolded protein response pathway have been found neurons in a triplication of the *SNCA* gene dosage^63^, and we previously reported that gene-correction of the A30P mutation rescues the mitochondrial interaction network^35^.

The importance of using isogenic patient-derived gene-corrected cell lines to model disease is that it enables assessment of the mutation-only effect in isolation from the variation within the genetic background that by itself may contribute to, or alternatively mask the mutation-specific phenotypes. However, there are still limitations within our cellular model, we find significant inter-clonal variability between our gene-corrected isogenic cell lines in terms of TH neuron generation, and neuronal markers, yet at the functional characterisation level these cell lines align. The typical efficiency to generate dopaminergic neurons for PD research is 10–40%^65–67^, although recent protocols have become more efficient^68^, these dopaminergic neurons are not “aged”^69^ and the remaining cell types within those iPS-derived cultures is unclear. In our extensive characterisation of our in vitro model at 30 days post-directed differentiation, we find a combination of neural progenitors, immature neurons, mature neurons as well as a small proportion of astrocytes. Simply put, modelling age-related neurodegenerative diseases remains challenging.

We have previously shown an upregulation in *SNCA* in the pathological neurons using qRT-PCR^30^, which we have confirmed using RNA-seq (Supp. Fig. 6). However, using an antibody specific for phosphorylated alpha-synuclein (Serine 129) that we validated in 70-day old midbrain organoids carrying a triplication of the *SNCA* locus^70^, we were not able to find evidence of phosphorylated alpha-synuclein in the pathogenic neurons that express physiological levels of endogenous alpha-synuclein (Supp. Fig. 6). It is unclear if this is due to, or a combination of: our 2D cellular model with limited aging capacity, our reduced percentage of dopaminergic neurons, or the neurons at this timepoint do not display phosphorylation at Serine 129 and/or have other prior post-translational modifications^71^.

Finally, we must consider that in the PD patient, physiological levels of pathogenic alpha-synuclein protein become symptomatic with catastrophic downstream consequences only later in life, with the onset age of the p.A30P *SNCA* mutation carriers ranging from 54 to 76 years old, which is closer to sporadic PD than the p.A53T *SNCA* or *SNCA* triplication locus carriers^31^. Nevertheless, the synaptic plasticity that previously compensated for the mutation-effect must decline during the aging process and contribute to the disease pathogenesis. For example, we find it interesting that under mitochondrial stress conditions the neurons carrying the p.A30P mutation can generate a maximum respiration no less than the isogenic controls, yet under basal conditions this does not occur leading to an energy deficit that will only accumulate over time. Our findings provide the first evidence of a reduction in energy and an aberrant lipid metabolism as causative mechanisms that underpin PD pathology, which either leads to, or correlates to a specific reduction in dopaminergic neuritic processes and reduced neuronal function. Furthermore, we have identified kinases and transcriptional regulators that can act as an entry point for targeted drug-screening strategies, and moreover, have found in pathogenic PD-neurons carrying the p.A30P *SNCA* mutation, upregulation of the alpha-synuclein interacting protein and early-stage PD biomarker *BRSK1*. Further validation studies will be required yet the exciting possibility of early-stage PD biomarkers can exert a profound clinical impact on the treatment of PD patients with potential treatment in the prodromal stage of the disease.

## Experimental procedures

### Subject material

A biopsy of dermal fibroblasts was donated with informed consent from a patient carrying the p.A30P mutation in *SNCA* at 67 years of age. The generation and characterisation of induced pluripotent stem cells (iPSCs) from the dermal fibroblasts has been described^34^ and has a unique identifier HIHDNDi001-B, also referred to as A30P-4. Another patient-derived iPS clone from this same individual has the unique identifier HIHDNDi001-A and is referred to as A30P-3^34^. An age- and gender-matched non-PD control iPS cell line, referred to as Control-1 has been previously published^35^, and a gender-matched non-PD control iPS cell line, referred to as Control-2 has been previously published^32^. The generation of single-cell gene-corrected patient-derived iPS clones from the A30P-4 iPS cell line obtained from the A30P patient has also been described^30^. Ethical approval for the development of and research pertaining to patient-derived cell lines have been given by the National Committee for Ethics in Research, Luxembourg (Comité National d’Ethique dans la Recherche; CNER #201411/05), and all methods were performed in accordance with the relevant guidelines and regulations.

### Maintenance of iPS, generation of NPCs and differentiation to vmDA neurons

The maintenance of iPSCs has been previously described^34^. The generation of ventral midbrain dopaminergic (vmDA) neurons was performed according to the protocol elsewhere described^67^. The vmDA neurons were generated via a multipotent neuronal precursor cell (NPC) stage. Each directed neuronal differentiation took place using NPCs with different passage numbers (10 > p < 30), where the neurons were expanded and directly differentiated for a minimum period of 30 days. The generation and characterisation of the NPCs and vmDA neurons used this study has been described with the protocol elsewhere repeated and made automation compatible for high-throughput drug screening^30,67,72^.

### Assessment of neuronal networks

After 28 days of directed neuronal differentiation, neurons were treated with pre-warmed Accutase (Sigma-Aldrich, St. Louis, MO, USA; A6964) for approximately 10 min and placed in an incubator (37 °C, 5% CO_2_). The neurons were dissociated to obtain a single-cell suspension before DMEM was added and the cells centrifuged (300*g*; 5 min). The neurons were plated at 200,000 cells/coverslip with the addition of 10 μM Rho-Kinase Inhibitor Y-27632 (10 μM; Abcam, Cambridge, UK; Ab120129). After 48 h, the neurons were fixed and stained as previously described^34^ using primary antibodies for β-3-Tubulin (TUJ1; 1:200; BioLegend, San Diego, CA, USA; 801201), TH (1:300; Millipore, Burlington, MA, USA; AB152), Map2 (1:500; Sigma-Aldrich, St. Louis, MO, USA; M4403), Nestin (1:500; R&D Systems, Minneapolis, MN, USA; MAB1259), GFAP (1:1000; Dako, Agilent, Santa Clara, CA, USA; Z0334), Vimentin (1:100, Santa Cruz Biotechnology Inc., Texas, USA; sc-373717), phosphorylated alpha-synuclein (Serine 129)(1:100; Cell Signaling Technology, Danvers, MA, USA; 23706). Secondary antibodies were used as previously described^30^. For the image acquisition, 5 to 12 Z-stack images per coverslip were acquired using Zeiss spinning disk confocal microscope from three-to-four independent directed neuronal differentiations. The custom image analysis algorithms for the morphological analysis of neurology morphology, neurite morphology and skeletonisation, nuclei segmentation and TH neuron segmentation have been previously described^32,33^.

### RT-qPCR

RT-qPCR was used as previously described using RNA extracted from d30 neurons^30^. Hydrolysis probes against *ACTB* (Hs03023880_g1), *TH* (Hs00165941_m1), *TUBB3* (Hs00801390_s1) and *GFAP* (Hs00909233_m1) were used with genes normalised to *ACTB*. Total RNA extracted from patient-derived fibroblasts were used as a negative control^31^. Each sample and primer set was run in triplicate and relative expression levels were calculated using the ΔΔCt method.

### Electrical recording using multi-electrode arrays

Neurons were dissociated after 45 days of directed differentiation and plated at 200,000 cells/well into a minimum of 4 wells of a 48-well (16 electrode) CytoView MEA (Axion Biosystems, Atlanta, GA, USA; M768-tMEA-48W). The neurons were cultured on the plate for an additional 10 days and recorded at day 55. Briefly, the MEA plate was recorded using the Axion Integrated Studio (AxIS) software, version 2.1 (Axion Biosystems, Atlanta, GA, USA) on the Axion Maestro (Axion Biosystems, Atlanta, GA, USA). The plate was recorded for 5 min at 37 °C and 5% CO_2_. The 5% CO_2_ was obtained from a compressed gas bottle (Linde, Munich, Germany) and was regulated to the MEA at 0.2 bar. Re-recording of the MEA was performed using AxIS, software version 2.5, with a Butterworth (200 Hz–3 kHz) filter. The Spike Detector program was used to measure activity, the Adaptive Threshold Crossing method was used to detect crossings at 6 × Standard Deviation. The neural metrics were analysed using the Neural Metric Tool, software version 2.6.1 (Axion Biosystems, Atlanta, GA, USA). The threshold for an active electrode was set to five spikes per minute.

### Oxygen consumption rate measurement

The detection of oxygen consumption rate (OCR) was determined using the Seahorse XFe96 Extracellular flux bioanalyzer (Agilent technologies, Santa Clara, CA, USA) according to manufacturer instructions with the experimental conditions previously described^32^. A mitochondrial stress test was applied using the toxins: oligomycin (1 μM; 75351), FCCP (250 nM; C2920) and rotenone and antimycin A (5 μM; 557368 and A8674) (all chemicals were acquired from Sigma-Aldrich, St. Louis, MO, USA). Neurons were seeded (80,000 cells/well) in with the perimeter wells avoided, therefore a maximum of 6 replicates per row were seeded, with at least 2 rows/cell line. A minimum of three independent biological replicates from independent directed differentiations were performed. Post-experiment, the plate was lysed using RIPA buffer and normalised to protein using a BCA assay. Post-normalisation, the experimental data was exported using the Seahorse XF Cell Mito Stress Test Report Generator. The Multi-File Seahorse XF Cell Mito Stress Test Report Generator was used to assess three biological replicates with statistical analysis performed in GraphPad Prism software version 8 (GraphPad Software Inc. La Jolla, CA, USA).

### Assessment of mitochondrial membrane potential

Prior to imaging, neurons were seeded after 21 days of directed differentiation (100,000 cells/well) in 96-well assay plates (Greiner Bio-One, Austria; 655087). Three days later they were incubated for 30 min in maturation medium supplemented with Tetramethylrhodamine, Ethyl Ester, Perchlorate (TMRE) (10 nM, Thermo Fisher Scientific, Waltham, MA, USA; T669) and MitoTracker Green FM (100 nM, Thermo Fisher Scientific, Waltham, MA, USA; M7514). After washing with PBS (Thermo Fisher Scientific, Waltham, USA; 14190144), neurons were incubated with maturation medium containing TMRE and Z-stacks were acquired at 60× magnification using a Yokogawa Cell Voyager 8000 high-content screening system. An in-house Matlab image processing pipeline was used to assess mean intensity of TMRE signal in mitochondria (as assessed by MitoTracker masking). 8 fields per well and a minimum of 2 wells per condition were assessed for each biological replicate. A minimum of three independent biological replicates from independent directed differentiations were performed. Data was analysed and plotted using ggpubr package in R.

### Assessment of mitochondrial superoxide

MitoSOX Red, a mitochondrial superoxide indicator (2 μM, Thermo Fisher Scientific, Waltham, MA, USA; M36008) was used according to the manufacturers conditions, and as previously described, by flow cytometry^32^. The amount of mitochondrial superoxide was normalised per unit of mitochondrial mass (by MitoTracker Green FM). Starvation treatment of the neurons refers to incubation of the neurons in the cell culture medium with only the N2 supplement and without B27 or the vmDA trophic factors previously described^67^. Mean fluorescence of the unstained cells were subtracted from values of all mean fluorescence to account for autofluorescence. Cells were analysed with the BD LSRFortessa cell analyzer (BD Biosciences, Franklin Lakes, NY, USA). FlowJo software (FlowJo for Mac, Version V10, 2019, Becton, Dickinson and Company, Franklin Lakes, NY, USA) was used for gating and processing of the samples.

### Assessment of neuronal viability

The CellTiter-Glo Luminescent Cell Viability Assay (Promega, Madison, WI, USA; G7570) was used according to the manufacturer instructions to determine cell viability based on the real-time quantification of ATP, which is directly proportional to the number of cells in the culture. Briefly, after 34 days of directed differentiation, the neurons were treated with Accutase for 10 min and seeded on a 384-well plate pre-coated 24 h earlier with Geltrex LDEV-Free Reduced Growth Factor Basement Membrane Matrix (Thermo Fisher Scientific, Waltham, MA, USA; A1413201). Neurons were seeded at 25,000 cells/ well and were maintained in the plate for 5 days before treatment. rotenone (Sigma-Aldrich, St. Louis, MO, USA; 557368) or DMSO was added using the Echo acoustic liquid handler (Labcyte Inc., San Jose, CA, USA) with a 16 h incubation. The total luminescence was normalised to the confluence percentage using the SpectraMax i3x Multi-Mode Microplate Reader i3 (Molecular Devices, San Jose, CA, USA) that was taken on the same day of experiment prior to the addition of rotenone or DMSO. The luminescence was measured using the Cytation 5 (Bio-Tek Instruments Inc., Agilent technologies, Santa Clara, CA, USA) with a minimum of eight technical replicates used per condition. The experiment was repeated following four independent neuronal differentiations.

### RNA sequencing and data analysis

RNA was extracted from neuronal cultures at day 45 of differentiation using the Qiagen RNeasy extraction kit (Qiagen, Hilden, Germany; 74104). RNA quality was assessed with an Agilent 2100 Bioanalyzer (Agilent, Santa Clara, CA, USA) and 200 ng of total RNA was subjected to ribosomal depletion using the QIAseq FastSelect rRNA removal (Qiagen; Hilden, Germany; 334386). cDNA libraries were constructed using the NEBNext Ultra II Directional RNA Library Prep Kit (New England BioLabs Inc., Ipswich, MA, USA; E7760). Library molarity was determined by measuring the library size (approximately 350 bp) using the Fragment Analyzer with the High NGS Fragment 1–6000 bp assay (Agilent, Santa Clara, CA, USA; DNF-474) and the library concentration (approximately 2 ng/µl) using the Infinite 200Pro (Tecan, Männedorf, Switzerland) and the Quant-iT HS Assay Kit (Thermo Fisher Scientific, USA; Q33232). The libraries were denaturated, diluted to 280 pM and sequenced as paired-end 100 bp reads on an Illumina NovaSeq6000 (Illumina, San Diego, CA, USA) with a sequencing depth of approximately 50 million clusters per sample. Library preparation and sequencing procedures were performed by the same individual and a design aimed to minimize technical batch effects was chosen. Read quality of RNA-seq data in fastq files was assessed using ngs-bits (v2020_06-4) to identify sequencing cycles with low average quality, adaptor contamination, or repetitive sequences from PCR amplification.

Data was processed using an in-house snakemake workflow^73^, available as a git repository https://git-r3lab.uni.lu/aurelien.ginolhac/snakemake-rna-seq (release v0.2.3, and singularity image v0.4). Raw read quality was assessed by FastQC (v0.11.9)^74^. Adapters are removed using AdapterRemoval (v2.3.1)^75^, with a minimum length of the remaining reads set to 35 bp. Reads were mapped to hg38 (GRCh38.p13) using STAR (v.2.7.4a)^76^, featureCounts from the R package Rsubread (2.2.2)^77^ was used to count reads. All counts > 10 were used for differential gene expression analysis using the R package DESeq2 (v1.28.1)^78^. Normalization in DEseq2 was done using apeglm (v.10.0)^79^. All FPKM were calculated using DESeq2 package. Pathway analysis on DEGs with false discovery rate < 0.05 and a minimum log2 fold change cut-off of ± 2 was performed using Ingenuity Pathway Analysis (IPA) tool. The functional analysis of RNA-seq data was done and networks were generated through the use of IPA (Qiagen Inc., Hilden, Germany; Content version: 60467501 (release date: 2020-11-19))^80^. Data was analysed and plotted using ggpubr package in R.

### Statistical analysis

Statistical analyses were performed with GraphPad Prism software version 8 (GraphPad Software Inc. La Jolla, CA, USA) or R^81^. The statistical analyses performed, and the P-value of each experiment can be found in the legend of the figures.

## Supplementary Information

Supplementary Information.
