# Supplementary material for: Gene-corrected p.A30P SNCA patient-derived isogenic neurons rescue neuronal branching and function
Source: Sci Rep. 2021 Nov 9;11:21946. doi: 10.1038/s41598-021-01505-x (PMC8578337; doi:10.1038/s41598-021-01505-x)
Supplement: Supplementary file 1 — Supplementary Information. [file 41598_2021_1505_MOESM1_ESM.pdf]

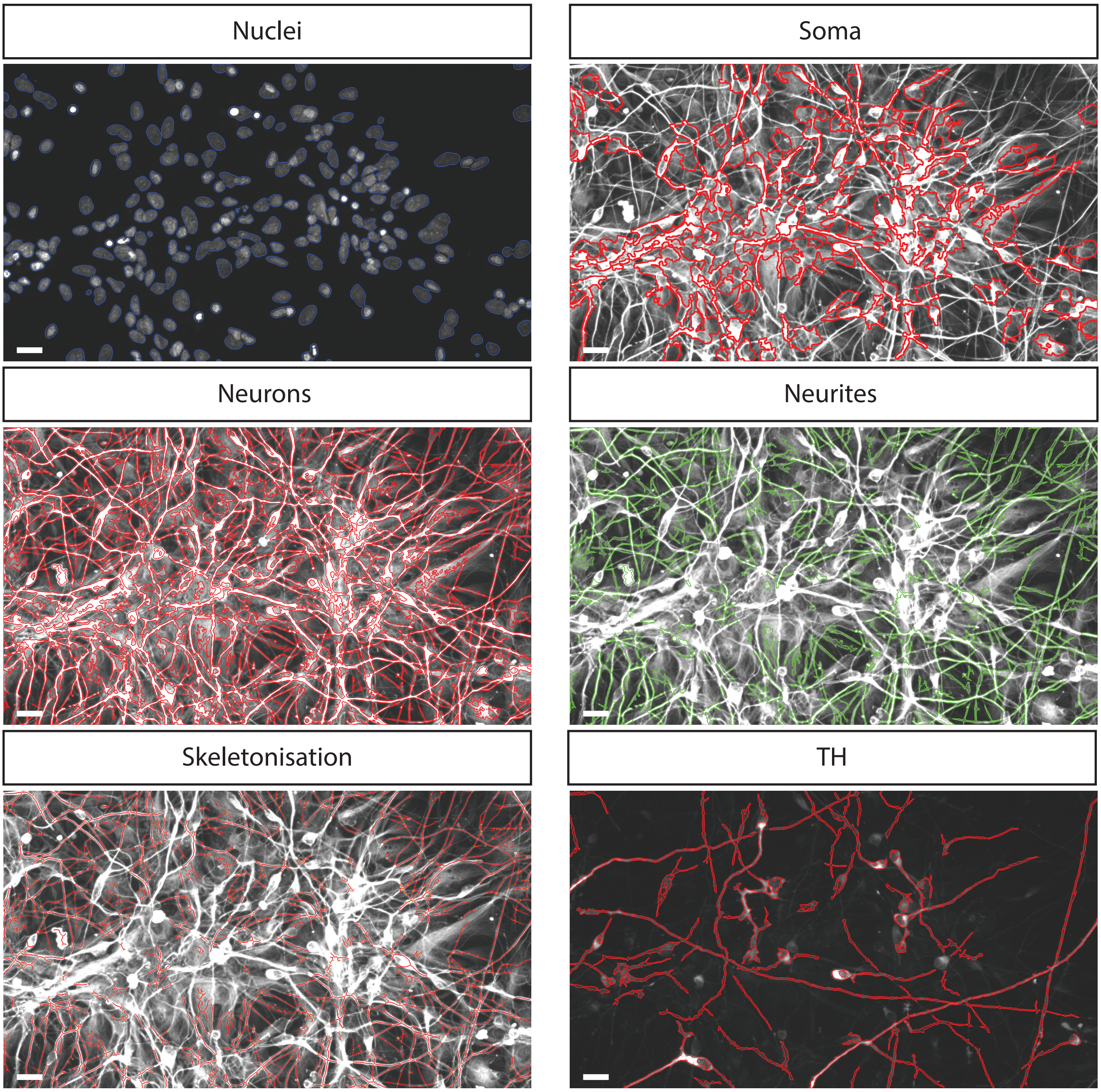

Supplementary Figure 1: Segmentation of neuronal network analysis. Representative image of a maximum intensity projection taken from a z-stack of d30 neurons fluorescently labelled with the neuronal marker Tuj1 and the dopaminergic marker TH.

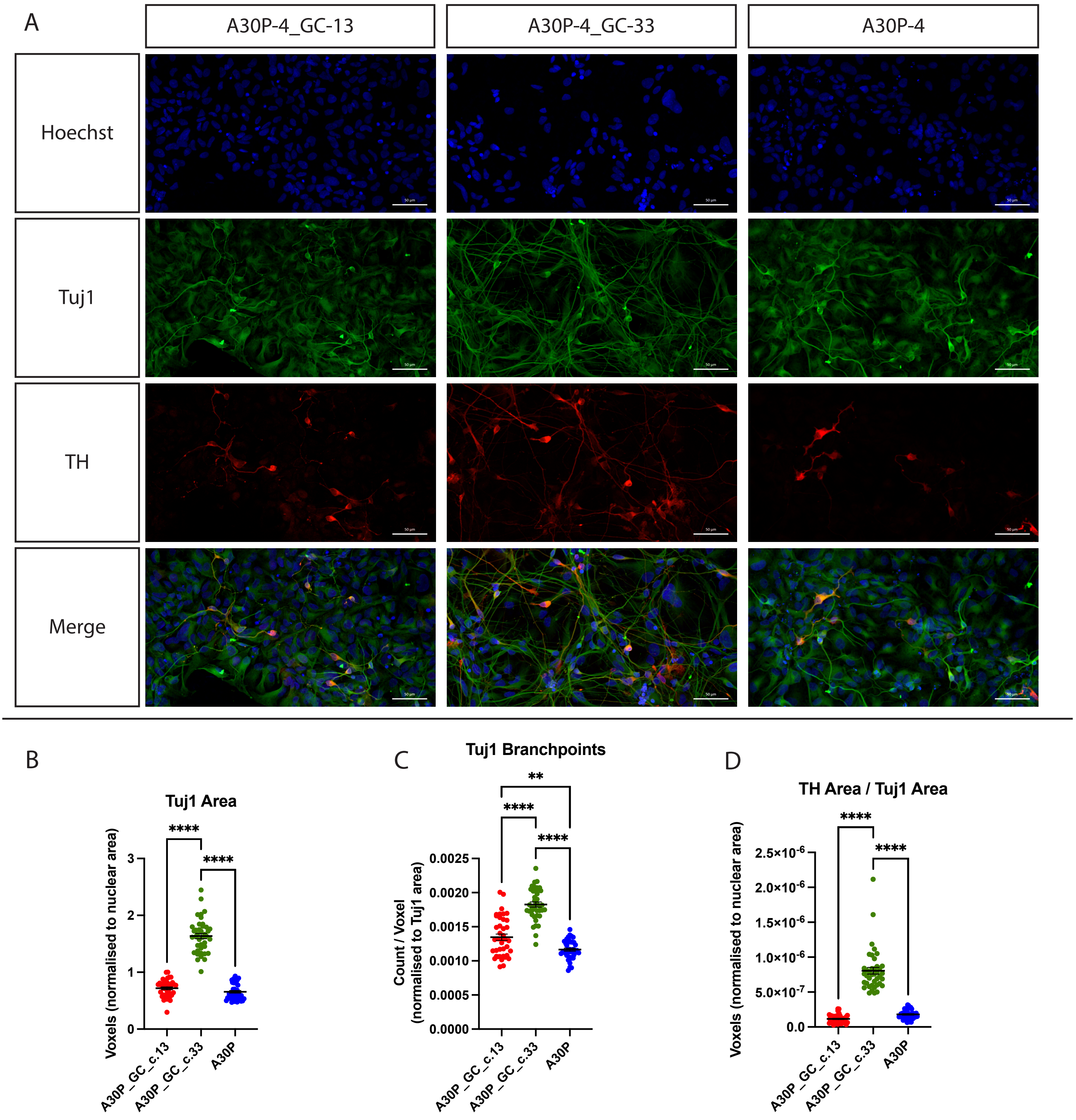

Supplementary Figure 2: Characterisation of d30 neurons fluorescently labelled with Tuj1 and TH antibodies. (A) Representative image of a maximum intensity projection acquired using a Z-stack for each cell line. (B) Quantification of Tuj1 area in the cellular population, (C) Tuj1 branchpoints, and (D) the proportion of TH / Tuj1labelled cells.

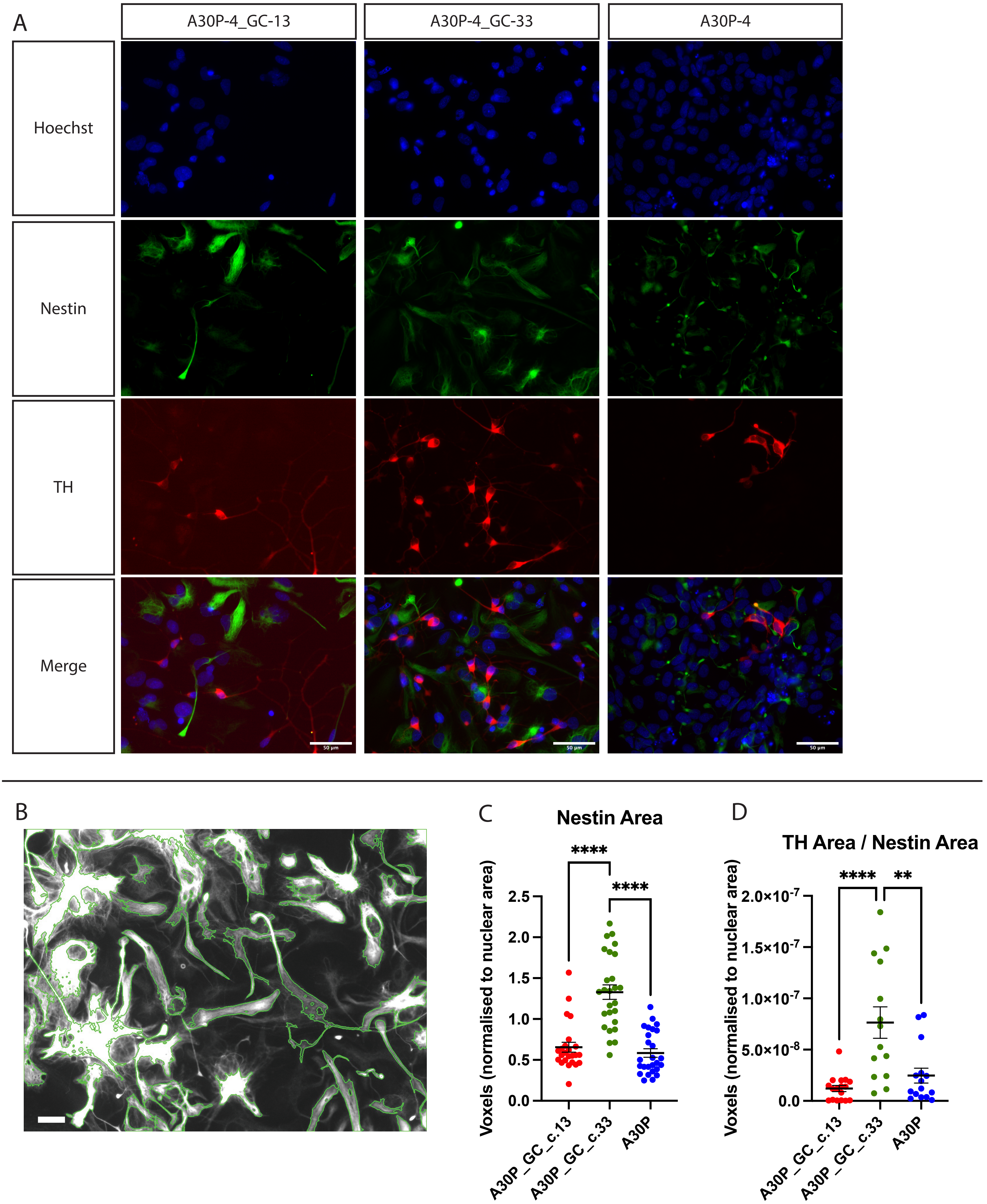

Supplementary Figure 3: Characterisation of d30 neurons fluorescently labelled with Nestin and TH antibodies. (A) Representative images of Nestin and TH antibodies (B) Representative image capturing the segmentation and labelling of Nestin positive cells. (C) Quantification of Nestin area in the cellular population and (D) the proportion of TH / Nestin labelled cells.

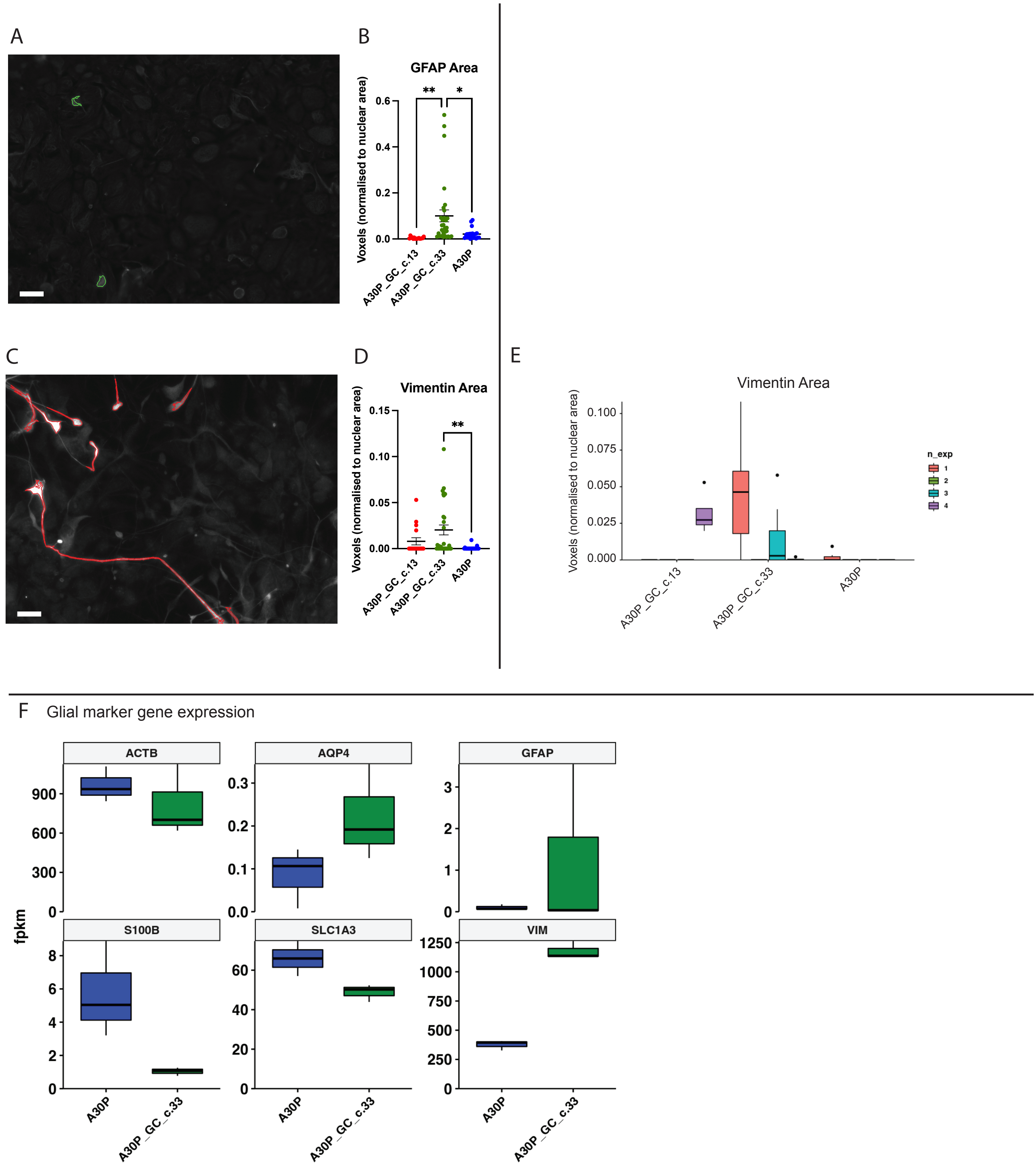

Supplementary Figure 4: Glial characterisation of d30 neurons. (A) Segmentation showing labelling for cells fluorescently labelled with an antibody specific for GFAP. (B) Quantification of GFAP Area. (C) Segmentation showing positive labelling for an antibody specific for Vimentin. (D) Quantification of Vimentin area (E) Variability in detectable levels of Vimentin across four independent neuronal differentiations. (F) Glial marker gene expression obtained from RNAseq.

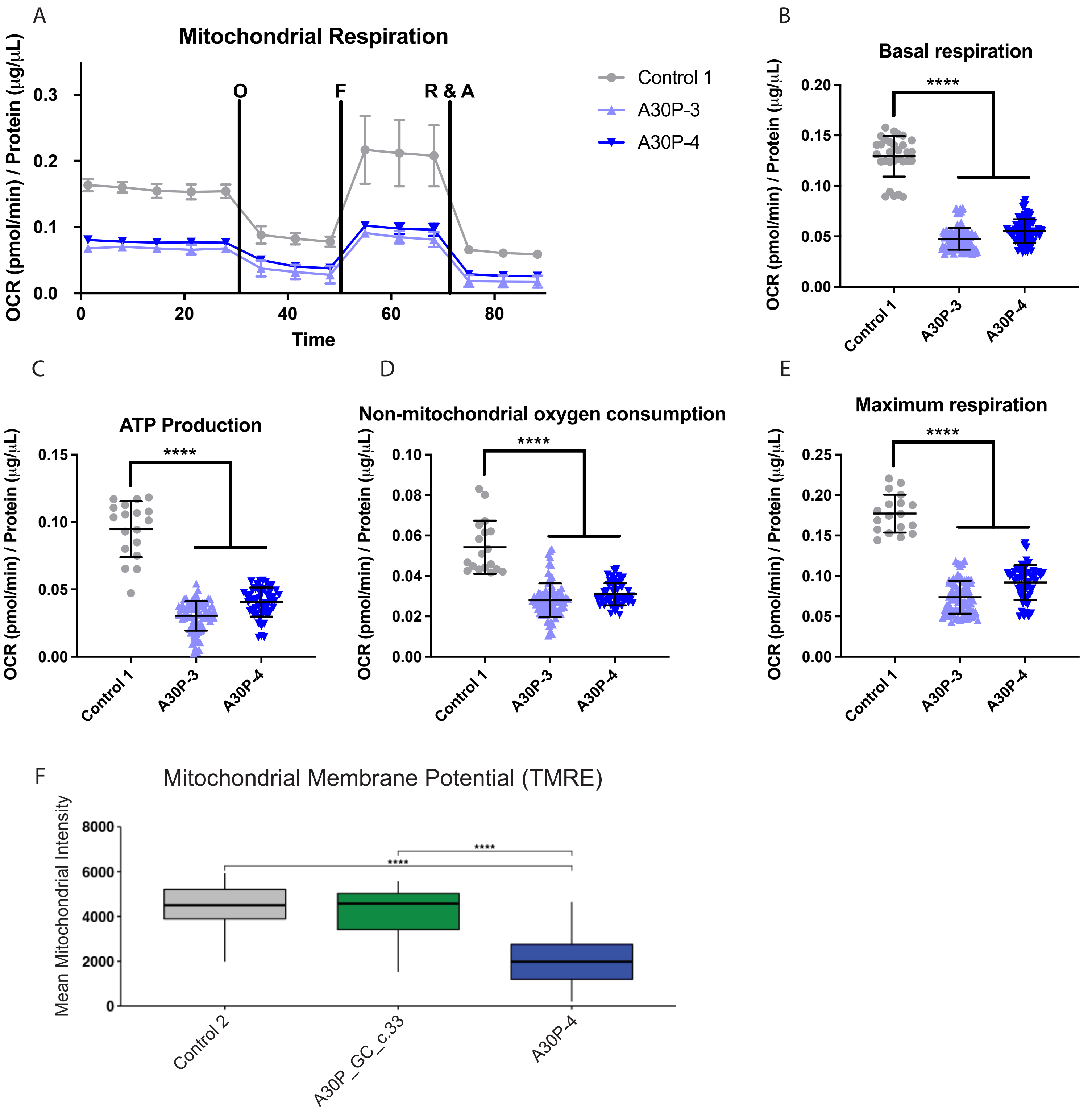

Supplementary Figure 5: Bioenergetic profile of patient-derived neurons following a mitochondrial stress test. Two iPS clones from the same individual harbouring the p.A30P mutation were differentiated and compared against an age- and gender-matched non-PD control cell line. (A) The cumulative bioenergetic profile (n=3) showing oxygen consumption rate (OCR) is shown at basal conditions and following a mitochondrial stress test with Oligomycin (O, 1uM), FCCP (F, 500nM) and Rotenone and Antimycin A (R & A, 10uM). The rates of (B) Basal respiration, (C) ATP production, (D) Non-mitochondrial oxygen consumption and (E), Maximum respiration are all shown. (F) Change in mitochondrial membrane potential was assessed using TMRE, comparing another gender-matched non-PD control, gene corrected clone 33 and the A30P-4 cell line. For multiple comparisons using statistical analysis (B-E), a one-way ANOVA was performed using Dunnett's post-hoc multiple-comparison test, (F) a non-parametric Kruskal-Wallis test was performed with a Dunn's post-hoc multiple comparison test . Figure (A) was plotted using mean +/- SEM, figures (B-F) were plotted as mean +/- SD. ****p<0.0001

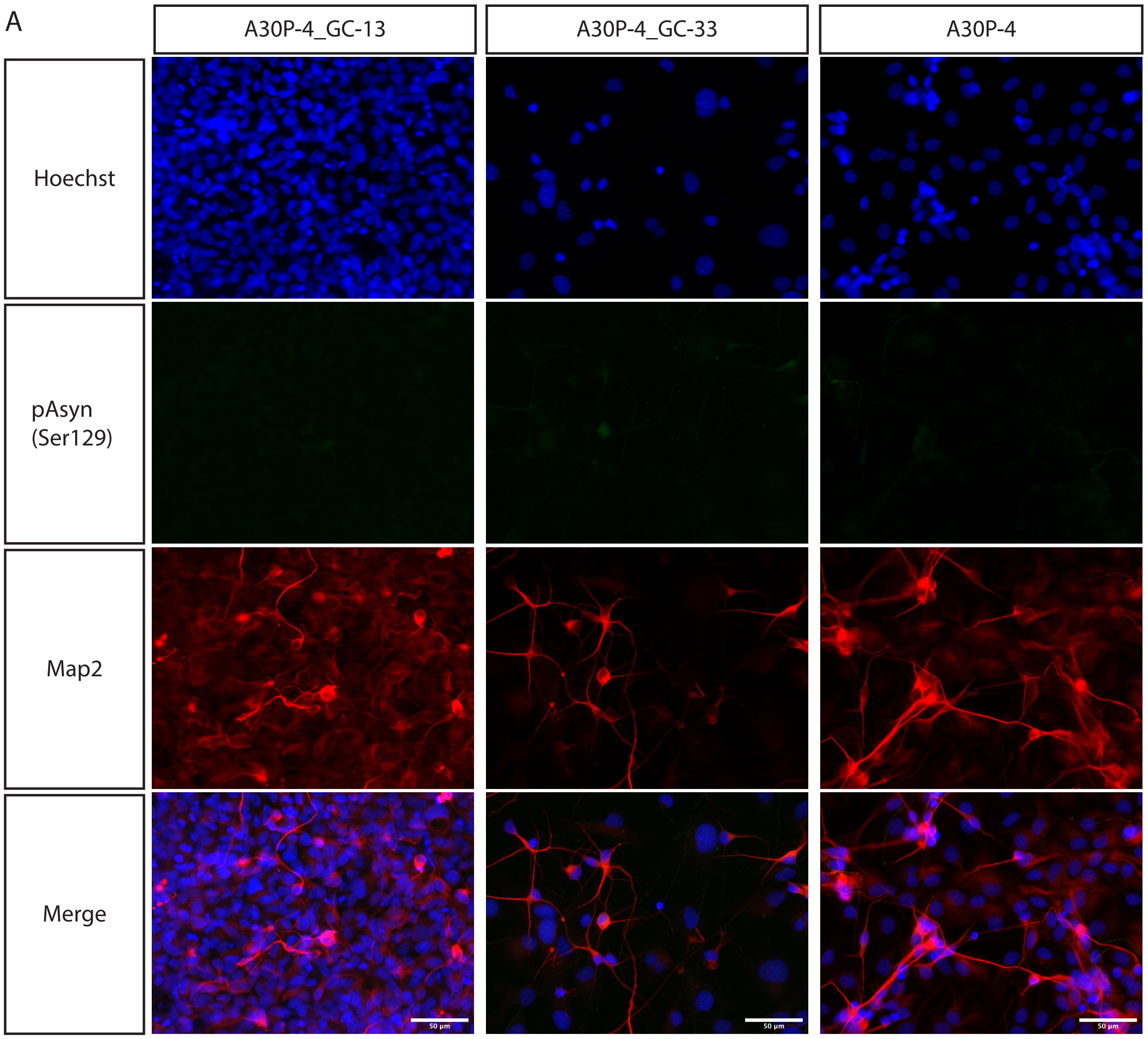

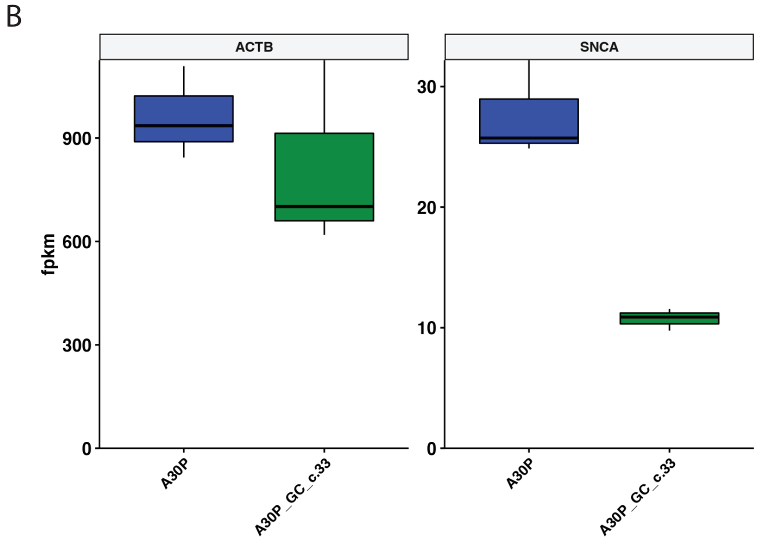

Supplementary Figure 6: Characterisation of alpha-synuclein. (A) Representative image of neurons fluorescently labelled with antibodies against phosphorylated alpha-synuclein (Serine 129) and Map2. (B) RNA-seq analysis based expression levels of SNCA and ACTB

A

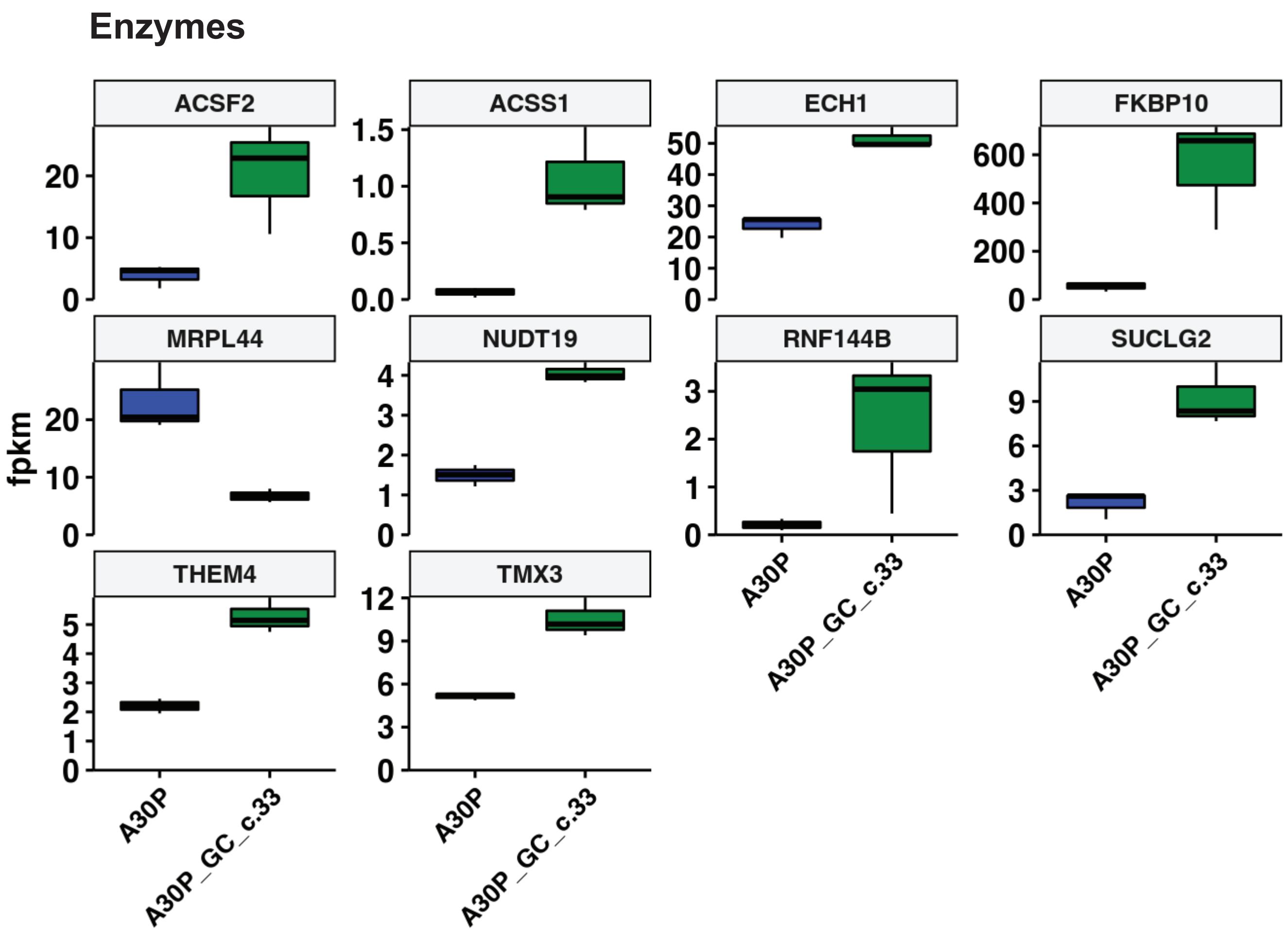
Enzymes
ACSF2
ACSS1
ECH1
FKBP10
MRPL44
NUDT19
RNF144B
SUCLG2
THEM4
TMX3
fpkm
A30P
A30P_GC_c.33

B

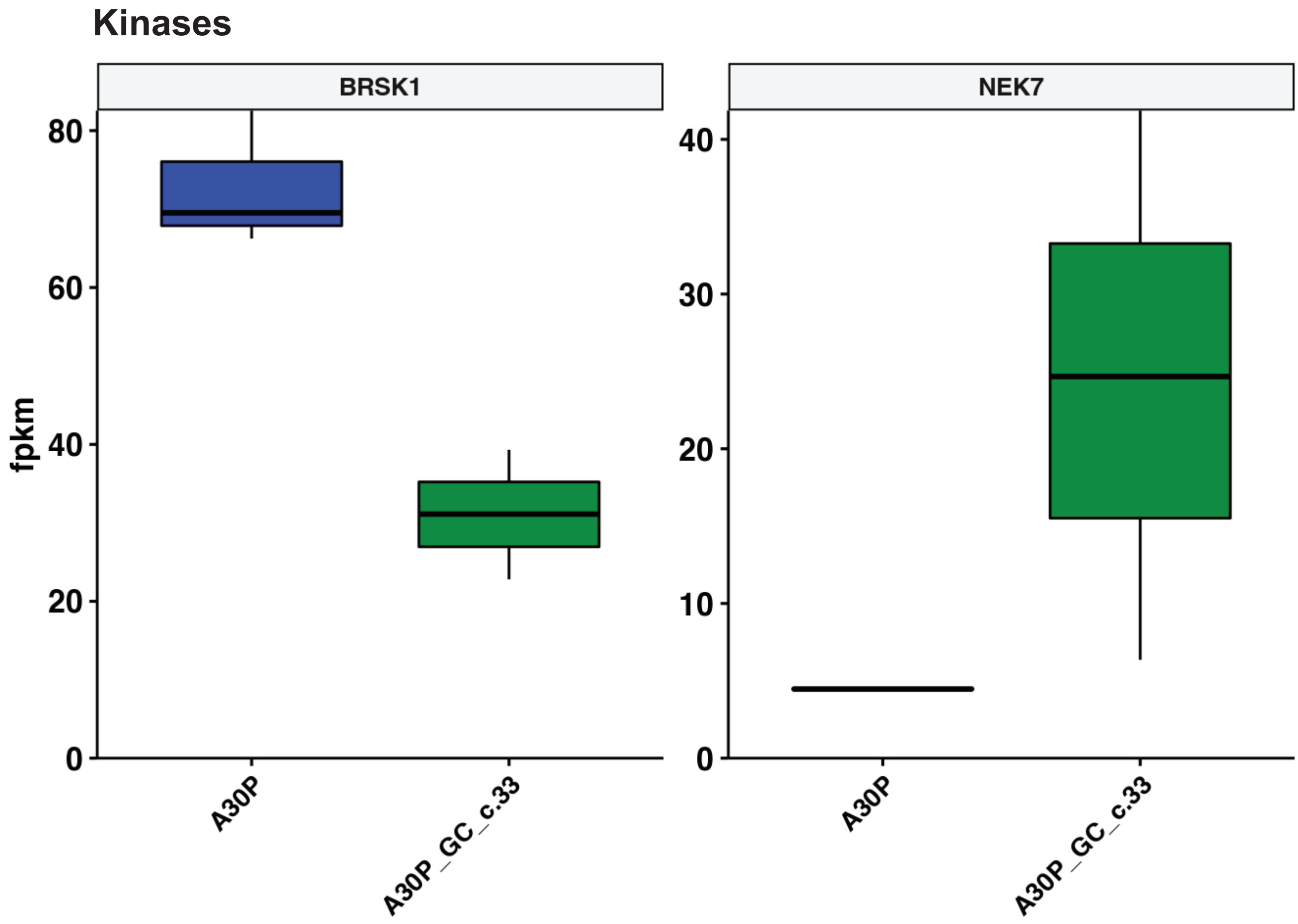
Kinases
BRSK1
NEK7
fpkm
A30P
A30P_GC_c.33

C Transcriptional Regulators

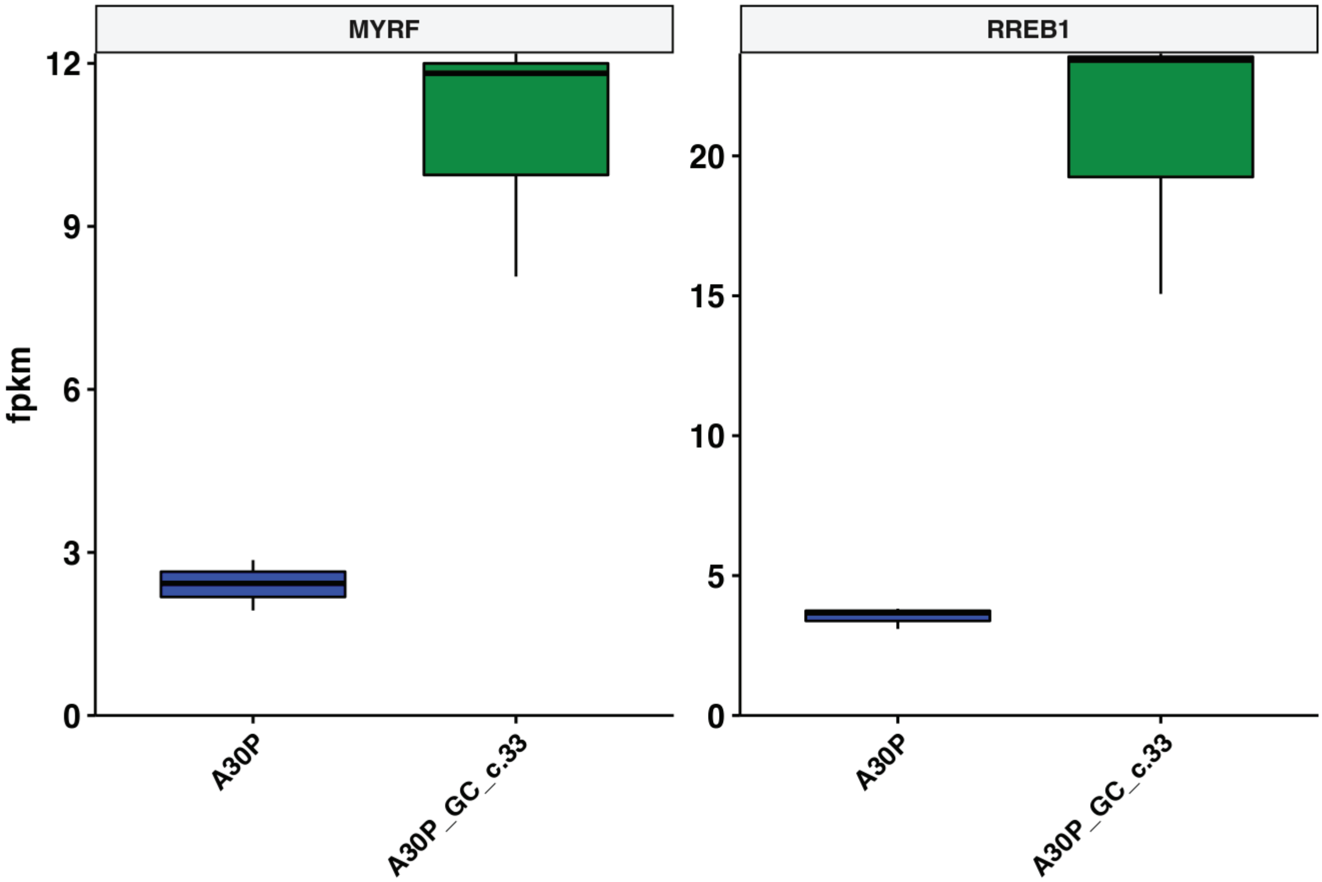

D Peptidases, Phosphatases and Ion Channel

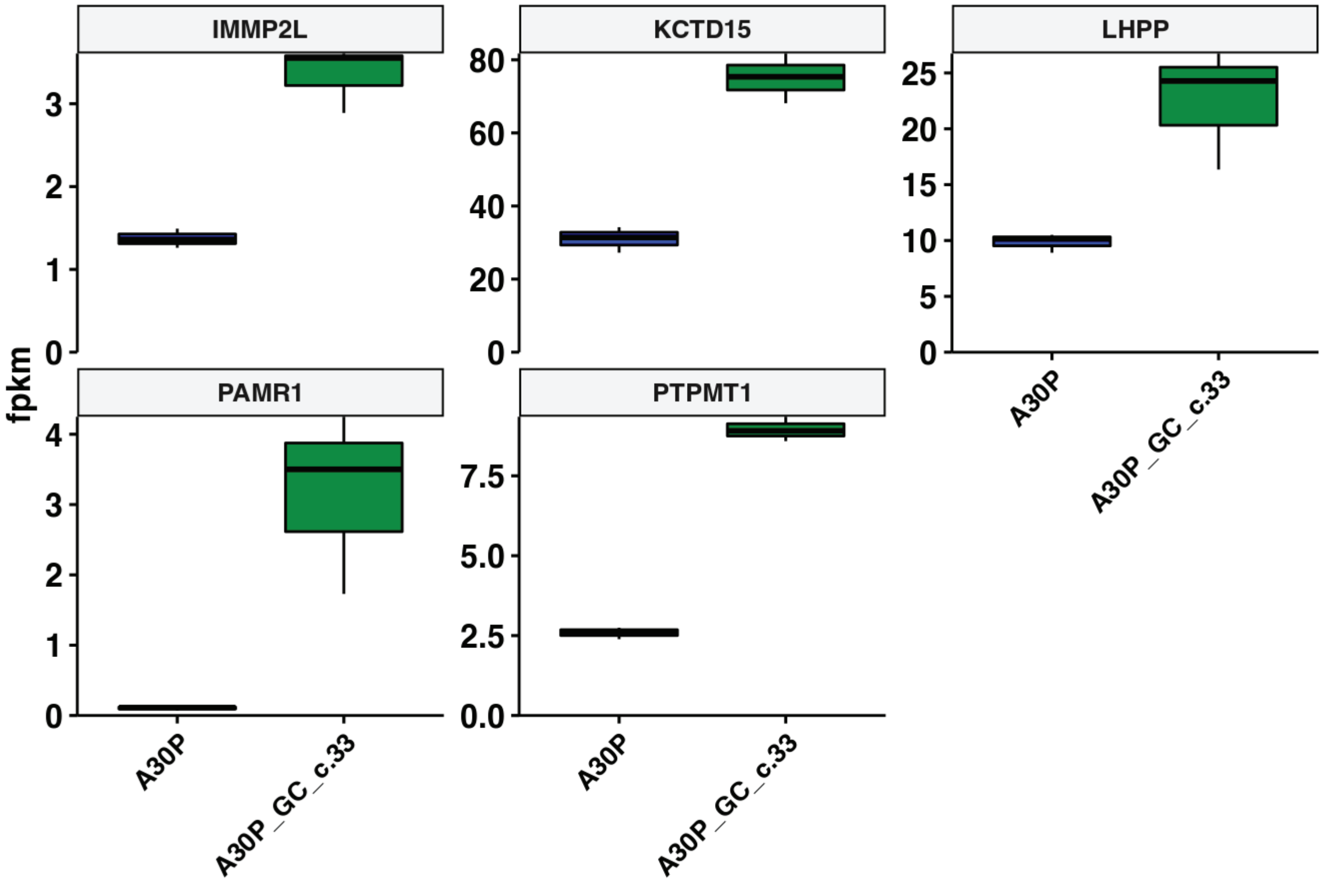

E

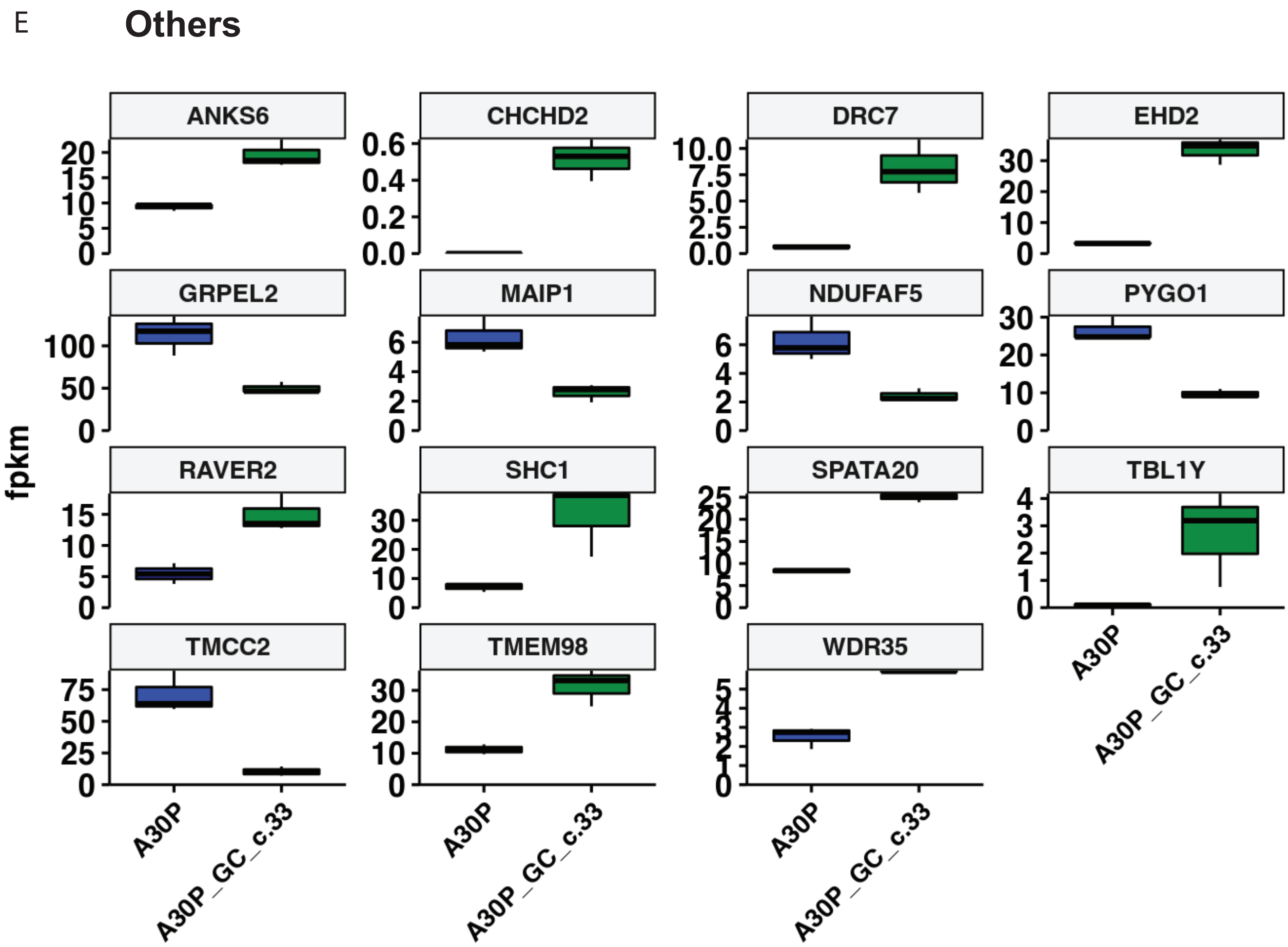

Supplementary Figure 7: Differential gene expression of lipid metabolism pathway genes. Genes are grouped into categories according to: (A) Enzymes, (B) Kinases, (C) Transcriptional Regulators, (D) Peptidases, Phosphatases and Ion Channel and (E) Others

Supplementary Tables

Supplementary Table 1: Clinical Assessment of the A30P patient in September 2010, 13 years of disease duration since diagnosis

Clinical assessment	Score
Age at the clinical assessment	68 years
Hoehn and Yahr scale	Stage 3
Mini-Mental State Exam (MMSE)	26 (equivalent to MoCA 22) [56]
Swab and England Activities of Daily Living Score	60%
Beck Depression Inventory (BDI) Version I total score	6
UPDRS I total score	1
UPDRS II total score	15
UPDRS IV total score	3
UPDRS III total score (ON medication, ON Stimulator)	28
UPDRS III total score (ON medication, OFF Stimulator)	57
UPDRS III total score (OFF medication, ON Stimulator)	35
UPDRS III total score (OFF medication, OFF Stimulator)	59
UPDRS III Medication ON/OFF (Stimulation ON) 28/35	amelioration of 20%
UPDRS III Stimulation ON/OFF (Medication ON) 28/57	amelioration 51%

Supplementary Table 2: Clinical diagnosis of the A30P patient motor and non-motor symptoms. Severity indicators: - absent; (+) discrete/variable; + mild; ++ moderate; +++ marked.

Motor Symptoms	Severity	Non-motor symptoms	Severity
**Bradykinesia**	++	**Subjective cognitive impairment**	-
**Rigidity**	++	**Anxiety**	-
**Resting tremor**	-	**Depression**	+
**Postural Instability**	++	**Hallucinations**	-
**L-DOPA response**	++	**Dysphagia**	++
**Motor fluctuations**	++		
**Dyskinesia**	-		
**Freezing of Gait**	+		
